# Targeting glutaminase and mTOR

**DOI:** 10.18632/oncotarget.5263

**Published:** 2015-08-26

**Authors:** Kazuhiro Tanaka, Takashi Sasayama, Eiji Kohmura

**Affiliations:** Department of Neurosurgery, Kobe University Graduate School of Medicine and Kobe University Hospital, Kobe, Japan

**Keywords:** glutaminase, mTOR, glioma

Comprehensive genomic and proteomic analyses demonstrate that there is nearly universal activation of the PI3K pathway in glioblastoma (GBM) patients [1]. Persistent PI3K signaling promotes GBM formation and tumor progression in genetic mouse models, establishing PI3K and its effecter mTOR as compelling molecular targets. mTOR, which has two distinct complexes, mTORC1 and mTORC2, is a protein kinase that integrates oncogenic signaling from growth factor receptors through PI3K, with cellular energy and nutrient status, to activate downstream signaling pathways that promote tumor growth and survival. Despite the perceived importance of mTOR as a molecular target in GBM, early-phase clinical trials using the mTOR inhibitor rapamycin failed to show efficacy. This result was potentially related to feedback activation of PI3K/Akt signaling and homeostatic regulation of other critical signals and cellular metabolism [2].

Oncogenic signaling pathways directly promote metabolic reprogramming to upregulate the biosynthesis of proteins, nucleotides, amino acids and lipids, required for the enhanced growth of cancer cells. These alterations include aerobic glycolysis known as the Warburg effect, which provides cancer cells selective advantages through enhanced catabolism of glutamine [3]. Glutamine is catabolized to α-ketoglutarate (αKG), an intermediate of the tricarboxylic acid (TCA) cycle, through two deamination reactions in a process termed glutamine anaplerosis. The first reaction requires glutaminase (GLS) to generate glutamate. The second reaction requires either glutamate dehydrogenase (GDH) or transaminases. Cancer cells usually display persistent TCA cycle activity despite robust aerobic glycolysis and often require mitochondrial catabolism of glutamine to TCA cycle intermediates to maintain rapid proliferation. Therefore, elucidating the role of glutamine metabolism could lead to significantly more effective targeted therapies for cancers.

We recently examined the role of glutamine metabolism in response to mTOR-targeted treatments for GBM [4]. Surprisingly, mTOR-targeted treatments affected metabolic reprogramming and increased GLS expression to deliver glutamine carbon to the TCA cycle, revealing that GBM cells were strongly addicted to glutamine. This compensatory glutamine metabolism allowed GBM cells to survive mTOR inhibitor treatment and was mediated by regulating the levels of αKG. The suppression of GLS by RNA interference or GLS inhibition with Compound 968 sensitized GBM cells to mTOR-targeted therapies. The combined mTOR and GLS inhibition caused synergic tumor cell death in mouse xenograft models.

These results demonstrate that the inhibition of mTOR signaling is sufficient to change the metabolic characteristics of GBM cells. Increasing GLS expression to elevate glutamate levels could be a method of compensating for the loss of TCA intermediates that occurs when mTOR inhibition reduces glycolysis. It is possible that mTOR inhibition may also induce the use of glutamine carbon sources to sustain the TCA cycle. Glutamate-derived αKG can be metabolized through the oxidative TCA cycle and/or reductively catabolized into isocitrate and citrate [5]. It will be interesting to employ metabolic flux analysis to investigate the source of metabolites immediately upstream and downstream of αKG in response to mTOR inhibition treatments.

Another open question concerns the functional link between mTORC1 and glutamine metabolism. A recent study demonstrated that mTORC1 inhibition decreases glutamine metabolism by upregulating SIRT4 expression to suppress GDH activity [6]. We found that GBM cells increase glutamine metabolism with elevated GLS expression after mTOR inhibition. Our data may suggest a different pathway because GBM cells used in the present study are resistant to mTOR inhibition. Another possible mechanism involves GLS regulation by ERK signaling, which shows feedback activation after PP242 treatment [7]. These findings suggest that GBM cells have developed additional routes toward the resistance to mTOR-targeted treatments.

GLS has two isozymes in humans: kidney-type glutaminase (KGA) and liver-type glutaminase (LGA), which are encoded by GLS and GLS2 genes, respectively [3]. GLS has been identified as an oncogene regulated by c-Myc expression, while GLS2 is a p53 target gene and functions in tumor suppression. GLS (KGA) has two splice variants that differ only in their C-terminal regions, with the longer form retaining the acronym KGA and the shorter form being called glutaminase C (GAC). Interestingly, GAC is upregulated in many cancer cells. We also confirmed that GLS, which has a detectable GAC form (unpublished data), was highly expressed in many clinical GBM samples compared with normal brain tissues. These enzymes may be attractive targets for GBM therapies.

GBM is the most common adult malignant primary brain tumor and is one of the most lethal cancers. The median survival for GBM patients is 12-18 months from the time of initial diagnosis despite surgery, radiation and chemotherapy. Therefore, new therapeutic approaches are desperately needed. Recent genomic and proteomic analyses have developed biomarker-driven strategies and potential “GBM-specific molecular targets” have been applied over the last several decades. However, drug resistance to this category of therapeutics has led to minimal clinical efficacy to date. Our study provides new insight into GBM resistance mechanisms to targeted therapies and offers a compelling rationale for the simultaneous inhibition of mTOR and GLS as a promising combination therapy for this challenging brain cancer.
